# IL-12 Mediates T-bet–Expressing Myeloid Cell–Dependent Host Resistance against *Toxoplasma gondii*

**DOI:** 10.4049/immunohorizons.2400029

**Published:** 2024-04-30

**Authors:** Madison L. Schanz, Abigail M. Bitters, Kamryn E. Zadeii, Dana Joulani, Angela K. Chamberlain, Américo H. López-Yglesias

**Affiliations:** * Department of Microbiology and Immunology, Indiana University School of Medicine–Terre Haute, Terre Haute, IN; † Department of Biology, Indiana State University, Terre Haute, IN

## Abstract

To defend against intracellular pathogens such as *Toxoplasma gondii*, the host generates a robust type 1 immune response. Specifically, host defense against *T*. *gondii* is defined by an IL-12–dependent IFN-γ response that is critical for host resistance. Previously, we demonstrated that host resistance is mediated by T-bet–dependent ILC-derived IFN-γ by maintaining IRF8^+^ conventional type 1 dendritic cells during parasitic infection. Therefore, we hypothesized that innate lymphoid cells are indispensable for host survival. Surprisingly, we observed that T-bet–deficient mice succumb to infection quicker than do mice lacking lymphocytes, suggesting an unknown T-bet-dependent–mediated host defense pathway. Analysis of parasite-mediated inflammatory myeloid cells revealed a novel subpopulation of T-bet^+^ myeloid cells (TMCs). Our results reveal that TMCs have the largest intracellular parasite burden compared with other professional phagocytes, suggesting they are associated with active killing of *T*. *gondii*. Mechanistically, we established that IL-12 is necessary for the induction of inflammatory TMCs during infection and these cells are linked to a role in host survival.

## Introduction

A rapid host-mediated type 1 immune response is indispensable for the clearance of intracellular pathogens and host survival. *Toxoplasma gondii* has been a critical tool in mechanistically defining type 1 immune responses (1, 2). Host immunity against *T*. *gondii* has been defined by a myeloid cell−derived IL-12 response that generates an IFN-γ response from CD4^+^ Th1 cells (3–6). By exploiting *T*. *gondii* as a model for intracellular pathogens, it has been demonstrated by our group and others that group 1 innate lymphocytes (ILC1s) and NK cells are additional critical innate sources of IFN-γ during acute parasite infection (7–10). IFN-γ is critical for activating myeloid cells and initiating their expression of IFN-γ–inducible genes, which are indispensable for *T*. *gondii* clearance (11–15). This crosstalk between myeloid cells and lymphocytes is essential for host immunity against intracellular pathogens.

The transcription factor T-bet is considered essential for the development and function of ILC1s, NK cells, and Th1 cells during *T*. *gondii* infection (7, 16–19). Moreover, the role of T-bet is classically considered to be the master regulator of lymphocyte-derived IFN-γ production (20, 21). However, we and others have recently demonstrated that T-bet is not required for *T*. *gondii*–mediated NK cell– and Th1 cell–derived IFN-γ (7, 16, 17). Despite T-bet–independent IFN-γ production, T-bet–deficient (*Tbx21*^−/−^) mice succumb to parasite infection significantly quicker than do mice lacking T and B cells (*Rag2*^−/−^), indicating an additional role for T-bet in innate immunity against *T*. *gondii*. Recently, our group demonstrated that T-bet–dependent ILC1-derived IFN-γ is vital for sustaining IRF8^+^ conventional type 1 dendritic cells (cDC1s) (7). Taken together, these data demonstrate that T-bet–dependent ILC1-derived IFN-γ is critical for myeloid cells and lymphocytes to work in concert to generate effective host immunity against *T*. *gondii* infection.

Therefore, we sought to determine whether ILC1s were necessary for host resistance during parasite infection. Unexpectedly, we observed that *Tbx21*^−/−^ mice succumb to *T*. *gondii* infection significantly quicker than both lymphocyte-deficient (*Rag2*^−/−^*γc*^−/−^) and wild-type (WT) mice. Thus, we hypothesized that T-bet–expressing myeloid cells mediate parasite elimination during acute *T*. *gondii* infection. Mechanistically, we determined that IL-12 is required for mediating a novel subpopulation of T-bet^+^CD11c^+^MHC class II (MHCII)^−^ myeloid cells (TMCs) during *T*. *gondii* infection. Taken together, our results identify a previously undescribed T-bet–dependent pathway required for myeloid cell–mediated parasite clearance and host survival during *T*. *gondii* infection.

## Materials and Methods

### Mice

C57BL/6, *Tbx21*^−/−^, and CD11c-Cre, Tbx21-Cre, *Tbx21*^flox/flox^, *Irf8*^flox/flox^, R26R-EYFP mice were obtained from The Jackson Laboratory (Bar Harbor, ME), and *Rag2*^−/−^*γc*^−/−^ mice were obtained from Taconic Biosciences (Rensselaer, NY). CD11c-Cre mice crossed with *Tbx21*^flox/flox^ or *Irf8*^flox/flox^ mice generated CD11c-*Tbx21*^−/−^and CD11c-*Irf8*^−/−^ mice, respectively. Tbx21-Cre crossed with R26R-EYFP mice generated Tbx21-EYFP mice. All mice were age and sex matched within individual experiments. All procedures were approved by the Institutional Animal Care and Use Committee of the host campus of Indiana University School of Medicine–Terre Haute, Indiana State University.

### Parasite maintenance and in vivo infection with *T. gondii*

Mice were i.p. infected with 20 cysts (ME49 strain) or 20,000 GFP-expressing tachyzoites (Prugniaud strain) of *T*. *gondii* as previously described (7). In some experiments, mice were injected i.p. with 300 ng of IL-12p70 (BioLegend) on days 0 and 1. In some experiments, mice were injected i.p. with 500 μg of anti–IL-12p40 (Bio X Cell) on days 0, 1, 2, and 3.

### Quantitative PCR

Total genomic DNA from animal tissue was isolated by using the DNeasy blood and tissue kit (Qiagen) according to the manufacturer’s instructions. PCR was performed using SsoFast EvaGreen supermix (Bio-Rad). Samples were measured by quantitative PCR (qPCR) using a MyiQ real-time PCR detection system (Bio-Rad), and data from genomic DNA were compared with a defined copy number standard of the *T*. *gondii* gene *B1* as previously described (7).

### Cytokine analysis

IL-12p40 concentration in the sera or peritoneal exudate fluid was analyzed by a standard sandwich ELISA kit according to the manufacturer’s instructions (Thermo Fisher Scientific).

### Flow cytometric analysis

To assay responses of mice infected with *T*. *gondii*, peritoneal exudate cells (PECs) were harvested from mice on days 0, 3, 5, or 8 postinfection (PI) by injecting 10 ml of collection solution (PBS with 5.0 mM EDTA) into the peritoneum (7, 18). To examine myeloid cell populations, a single-cell suspension of PECs was collected. All single-cell suspensisons were resuspended in cell culture media (RPMI 1640) and 5 × 10^6^ cells/well were plated. Cells were stained with Zombie Yellow (BioLegend) to assess viability. To surface stain for myeloid cells, the following fluorochrome-conjugated Abs were used: CD45, CD3, CD19, NKp46, Ly6G, CD11b, Ly6C, MHCII, F4/80, and CD11c. Intracellular staining of T-bet and *T*. *gondii* (p30) was performed by permeabilizing cells overnight at 4°C with a Foxp3/transcription factor staining buffer set (Thermo Fisher Scientific) according to the manufacturer’s instructions. A complete list of the clone and labeled fluorescence of the Abs is available upon request to the corresponding author. Gating strategy can be found in Supplemental Figs. 1A and 3. Fluorescence was measured using an LSR II flow cytometer (BD Biosciences) or Aurora flow cytometer (Cytek Biosciences), and data were analyzed using FlowJo software (version 10; Tree Star).

### Statistical analysis

All data were analyzed with Prism (version 9; GraphPad). These data were considered statistically significant when *p* values were <0.05. Error bars on all figures are representative of the SEM.

## Results

### *T. gondii* infection mediates the recruitment of a novel subpopulation of T-bet–expressing CD11c^+^ myeloid cells

**FIGURE 1.**
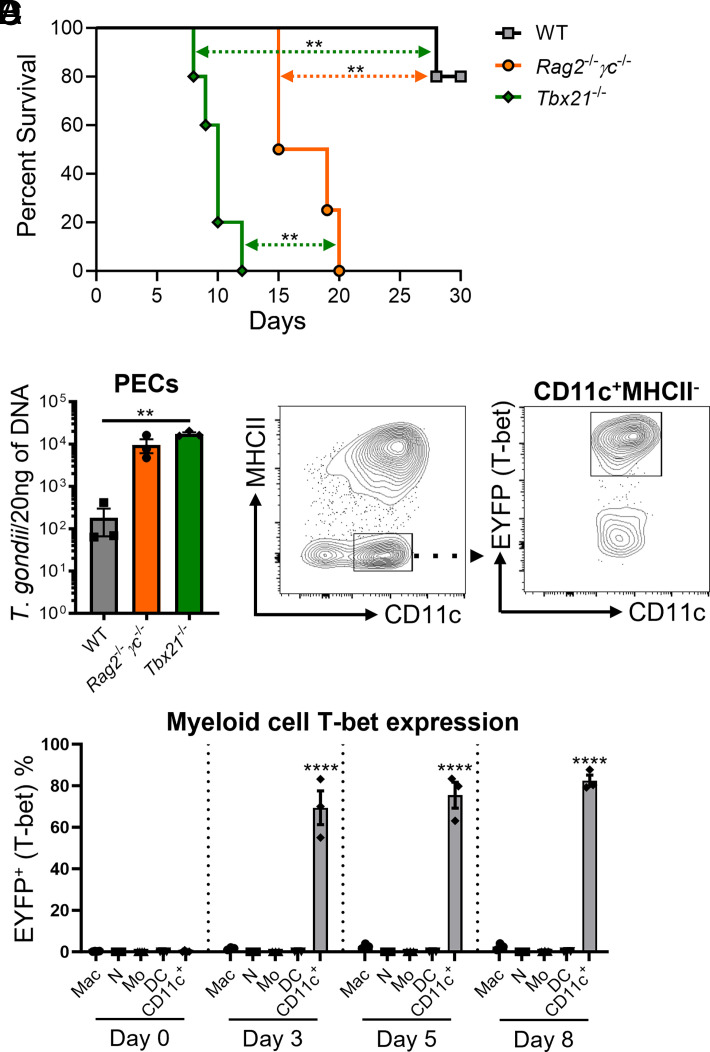
*T*. *gondii* infection mediates the recruitment of a novel subpopulation of T-bet-expressing CD11c^+^ myeloid cells. (**A**) Survival of WT (*n* = 5), *Rag2^−/−^γc*^−^*^/^*^−^ (*n* = 4), and *Tbx21*^−/−^ (*n* = 6) mice that were infected i.p. with 20 cysts. (**B**) WT, *Rag2^−/−^γc^−/−^*, and *Tbx21*^−/−^ mice were i.p. infected with 20 cysts and parasite burden was assessed from PECs of mice on day 8 PI by qPCR. (**C** and **D**) Tbx21-EYFP mice were infected i.p. with 20 cysts and peritoneal myeloid cells were evaluated for EYFP expression. (C) Representative contour plots on day 5 PI of CD3^−^CD19^−^NKp46^−^ (lineage [Lin]) Ly6G^−^Ly6C^lo^CD11c^+^MHCII^−^ cells and their EYFP expression. (D) Average frequencies of Lin^−^EYFP^+^ macrophages (Mac), neutrophils (N), monocytes (Mo), DCs, and CD11c^+^MHCII^−^ myeloid cells (CD11c^+^) at days 0, 3, 5, and 8 PI. Results are representative of three independent experiments involving at least three mice per group. Error bars indicate SEM. Statistical analyses were done using a log-rank (Mantel–Cox) test (A) or one-way ANOVA with a post hoc Tukey multiple comparison test between each group (B and D), **p <* *0.01, ****p <* *0.0001.

Based on our studies and those of others we predicted that ILC1s are necessary for host resistance against *T*. *gondii* (7, 10). Notably, we observed that *Tbx21*^−/−^ mice succumb to parasite infection significantly quicker than do *Rag2*^−/−^*γc*^−/−^ mice (Fig. 1A). We also observed that the lack of T-bet resulted in parasite burden comparable to *Rag2*^−/−^*γc*^−/−^ mice (Fig. 1B). Taken together, these data suggest a mechanism of T-bet–dependent myeloid cell–mediated immunity that is critical for host defense.

Myeloid cells such as macrophages, monocytes, neutrophils, and DCs have been established as indispensable for host immunity against *T*. *gondii*. However, there are limited studies investigating the role of T-bet in macrophages, monocytes, and neutrophils (Supplemental Table I). Therefore, we analyzed T-bet expression across these myeloid cell populations during *T*. *gondii* infection. To assess T-bet expression in myeloid cells, we generated a T-bet reporter mouse strain using Tbx21-Cre × R26R-EYFP (Tbx21-EYFP) mice. On days 0, 3, 5, and 8 following *T*. *gondii* infection, we observed no significant difference in the EYFP (T-bet) expression from peritoneal macrophages, monocytes, and neutrophils compared with cells from uninfected animals (Fig. 1D, Supplemental Fig. 1A**–**C), suggesting that T-bet plays a limited role in macrophage-, monocyte-, and neutrophil-mediated host defense against *T*. *gondii*.

In addition to the known role of macrophages, monocytes, and neutrophils during *T*. *gondii* infection, it has also been well established that DCs are required for host defense against acute parasite infection. We and others have demonstrated that cDC1-derived IL-12 is indispensable for mediating a rapid and protective IFN-γ immune response during infection (7, 22, 23). Notably, there are no current reports demonstrating that T-bet^+^ DCs play a role in myeloid cell–mediated host defense against intracellular pathogens. Studies have shown that T-bet expression in DCs plays a role in controlling inflammatory arthritis, priming of Ag-specific T cells, CpG DNA adjuvancy, and IFN-γ and TNF production (Supplemental Table I) (24–27). However, we observed very limited EYFP expression in peritoneal DCs from infected Tbx21-EYFP mice (Supplemental Fig. 1A**–**C). Interestingly, we observed a unique nonneutrophilic subpopulation of T-bet–expressing CD45^+^Lin^−^F4/80^−^Ly6C^lo^Ly6G^−^CD11c^+^MHCII^−^ (CD11c^+^MHCII^−^) myeloid cells on days 3, 5, and 8 following *T*. *gondii* infection in both the peritoneum and spleen. (Fig. 1C, 1D, Supplemental Fig. 1A, 3A).

To determine whether *T*. *gondii*–mediated CD11c^+^MHCII^−^ cells are derived from a myeloid origin, we used the *Rag2*^−/−^*γc*^−/−^ mouse model, which retains myeloid cells and lacks all lymphocytes. Both *Rag2*^−/−^ and *Rag2*^−/−^*γc*^−/−^ mice retained CD11c^+^MHCII^−^ cells during parasite infection (Supplemental Fig. 1D), confirming that CD11c^+^MHCII^−^ cells are not of lymphoid origin. Next, using Tbx21-EYFP mice, we examined whether these cells upregulated T-bet during infection. Markedly, CD11c^+^MHCII^−^ cells from infected Tbx21-EYFP mice displayed significant upregulation of EYFP on days 3, 5, and 8 PI compared with macrophages, monocytes, neutrophils, and DCs (Fig. 1D). Collectively, our experiments identified a novel *T*. *gondii*–mediated subpopulation of T-bet^+^CD11c^+^MHCII^−^ myeloid cells (TMCs) during acute infection.

### IL-12 is sufficient and necessary for maintaining inflammatory TMCs during *T. gondii* infection

**FIGURE 2.**
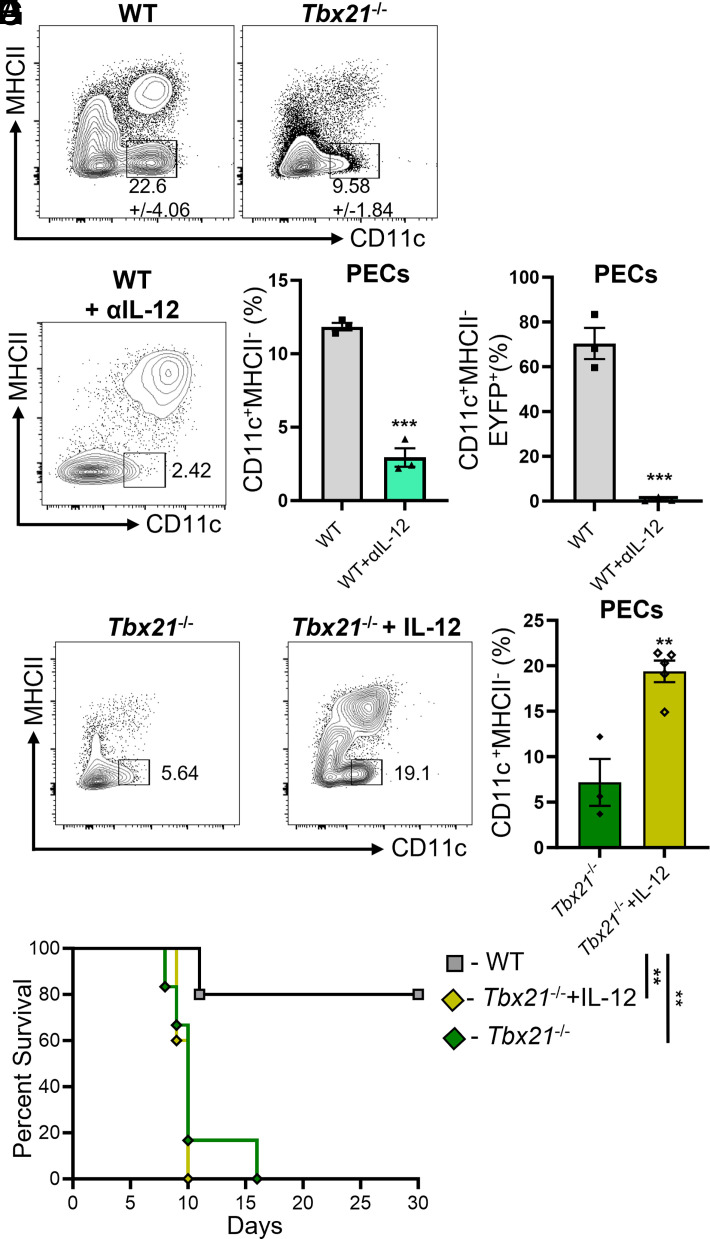
IL-12 is critical for regulating T-bet–dependent TMCs during *T*. *gondii* infection. (**A**) Representative contour plots of TMCs from the PECs of WT and *Tbx21^−/−^* mice infected with 20 cysts on day 5 PI. (**B–D**) Tbx21-EYFP mice were i.p. infected with 20 cysts and then treated with anti–IL-12p40 Abs during infection. (A and C) Representative contour plots and average frequencies of peritoneal TMCs on day 5 PI. (D) Representative contour plots and average frequencies of EYFP expression from (B). (**E–G**) *Tbx21*^−/−^ mice were i.p. infected with *T*. *gondii* and treated with or without rIL-12. (E and F) Representative contour plots and average frequencies of peritoneal TMCs on day 5 PI. (G) Survival of *Tbx21*^−/−^ mice administered IL-12 (*n* = 5), WT (*n* = 5), and *Tbx21^−/−^* (*n* = 6) control mice were i.p. infected with *T*. *gondii*. Results are representative of three independent experiments involving at least three mice per group. Error bars indicate SEM. Statistical analyses were done using an unpaired *t* test (C, D, and F) or log-rank (Mantel–Cox) test (G), **p <* *0.01, ***p <* *0.001.

Next, we examined whether T-bet is required for maintaining TMCs during parasite infection. We assessed the PECs from WT and *Tbx21*^−/−^ mice for TMCs following *T*. *gondii* infection, and in the absence of T-bet, TMCs are nearly undetectable on days 3, 5, and 8 PI (Fig. 2A and data not shown), suggesting that TMCs are T-bet–dependent. We proceeded to evaluate whether T-bet–dependent IFN-γ is required for unimpaired IL-12 production during *T*. *gondii* infection. In agreement with our previous report (7), we confirm that T-bet is critical for robust IL-12 production in both the sera and peritoneum during *T*. *gondii* infection (Supplemental Fig. 2A). To test whether IL-12 is required to maintain TMCs during *T*. *gondii* infection, mice were injected with IL-12–neutralizing Abs prior to parasite infection. Our data revealed that neutralizing IL-12 resulted in a significant reduction of TMCs on day 5 PI (Fig. 2B, 2C). Additionally, IL-12 neutralization led to a complete loss of EYFP expression in TMCs (Fig. 2D).

We next investigated whether cDC1s, a critical source of IL-12, are necessary to sustain inflammatory TMCs during infection. To determine the role of cDC1s in mediating TMCs during parasite infection, the CD11c-Cre system was used to conditionally delete IRF8-dependent cDC1s (CD11c-*Irf8*^−/−^). Infected CD11c-*Irf8*^−/−^ showed a significant reduction of TMCs on day 5 PI (Supplemental Fig. 2B), indicating that cDC1s are important to sustain inflammatory TMCs during *T*. *gondii* infection.

To further test our hypothesis that IL-12 is required to maintain TMCs during *T*. *gondii* infection, we analyzed the frequency of TMCs of infected *Tbx21*^−/−^ mice following administration of recombinant IL-12 (rIL-12). Our data showed that IL-12 administered to infected *Tbx21*^−/−^ mice rescued inflammatory TMCs at the site of infection (Fig. 2E, 2F). Despite IL-12 administration rescuing TMCs, accelerating NK-derived IFN-γ kinetics, restoring cDC1s, and reducing parasite burden in infected *Tbx21*^−/−^ mice (Fig. 2E, 2F, Supplemental Fig. 2C**–**F), it was insufficient to prevent their rapid mortality during *T*. *gondii* infection (Fig. 2G), suggesting that intrinsic T-bet expression by TMCs plays a role in host survival during parasite infection. Taken together, these data suggest that IL-12–dependent TMCs play a necessary role in host resistance against *T*. *gondii*.

### TMCs are critical for killing *T. gondii*

**FIGURE 3.**
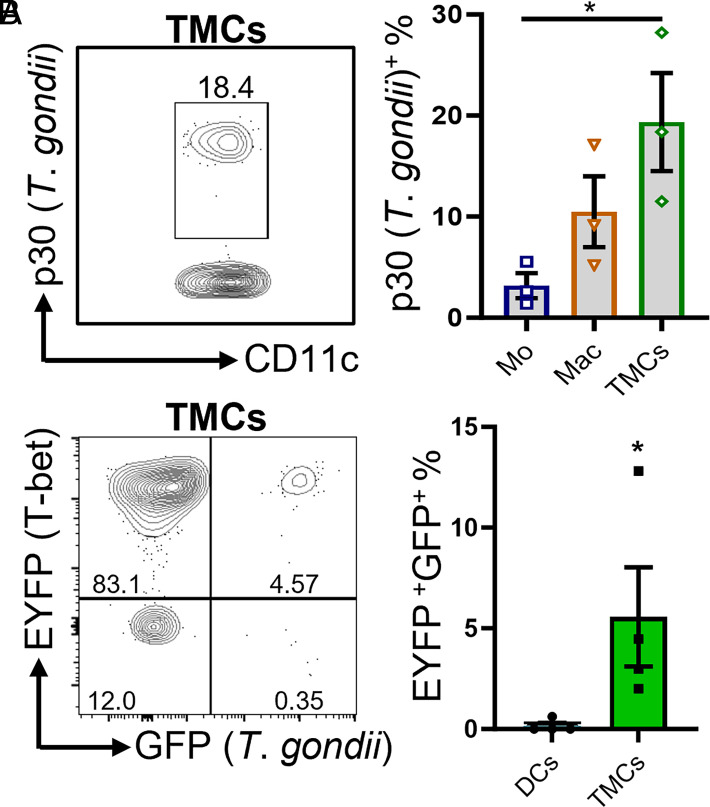
TMCs kill intracellular *T*. *gondii.* (**A**) WT mice were i.p. infected with 20 cysts. A representative contour plot and average frequencies of intracellular *T*. *gondii* (p30) in monocytes, macrophages, and TMCs from the peritoneum on day 5 PI are shown. (**B**) Tbx21-EYFP mice were i.p. infected with 20,000 *Tg*-GFP tachyzoites. A representative contour plot and average frequencies of intracellular *Tg*-GFP in DCs and TMCs on day 5 PI are shown. Results are representative of three independent experiments involving at least three mice per group. Error bars indicate SEM. Statistical analyses were done using one-way ANOVA with a post hoc Tukey multiple comparison test between each group (A) or unpaired *t* test (B), *p <* *0.05. Tg-GFP, *T*. *gondii* strain that constitutively expresses GFP.

Our results indicate that TMCs are critical for host defense against parasite infection, suggesting that they play a critical role for *T*. *gondii* clearance. We hypothesized that TMCs mediate parasite elimination during acute infection. We observed that TMCs displayed the highest frequency of intracellular *T*. *gondii* when compared with other professional phagocytes such as monocytes, macrophages, and DCs (Fig. 3A and data not shown), suggesting that TMCs are critical for *T*. *gondii* elimination. To demonstrate that TMCs are not more frequent than other cell types, making them more likely to be infected, we assessed the frequency of cells infected for each subset and observed that TMCs represent 0.72% of infected cells from the peritoneum on day 5 PI (Supplemental Fig. 3B).

To distinguish whether T-bet expression is associated with *T*. *gondii*–infected TMCs, we used a *T*. *gondii* strain that constitutively expresses GFP. Notably, most parasite-infected TMCs from Tbx21-EYFP mice are GFP^+^EYFP^+^ double positive (Fig. 3B). Additionally, we observed that most parasite-infected DCs are not associated with T-bet expression (Fig. 3B). Taken together, these results suggest TMCs could significantly contribute to the clearance of *T*. *gondii* during acute infection.

### Intrinsic T-bet expression by TMCs plays a role in host resistance against *T. gondii*

**FIGURE 4.**
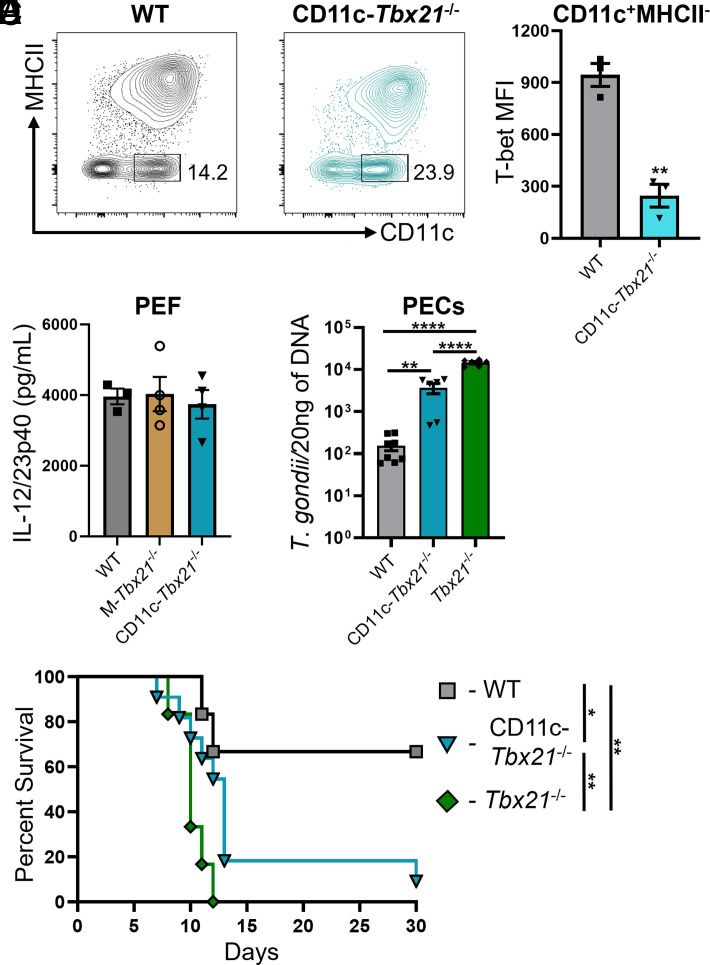
Intrinsic T-bet expression by TMCs is necessary for host resistance against *T*. *gondii.* (**A** and** B**) WT and CD11c-*Tbx21^−/−^* mice were i.p. infected and PECs were harvested on day 5 PI and TMCs were analyzed by flow cytometry. (A) Representative contour plots of TMCs and (B) the mean fluorescence intensity (MFI) of TMCs. T-bet expression in the PECs was analyzed on day 5 PI. (**C**) IL-12/23p40 analysis by ELISA of peritoneal exudate fluid (PEF) in WT, M-*Tbx21^−/−^*, and CD11c-*Tbx21^−/−^* mice following *T*. *gondii* infection on day 5 PI. (**D**) Parasite burden was assessed from the PECs in *T*. *gondii*–nfected WT, CD11c-*Tbx21^−/−^*, and *Tbx21*^−/−^ mice on day 8 PI by qPCR. (**E**) Survival curve of WT (*n* = 6), CD11c-*Tbx21^−/−^*(*n* = 6), and *Tbx21^−/−^* (*n* = 6) mice infected with 20 cysts. Results are representative of three independent experiments involving at least three mice per group. Error bars indicate SEM. Statistical analyses were done using an unpaired *t* test (B), one-way ANOVA with a post hoc Tukey multiple comparison test between each group (C and D), or log-rank (Mantel–Cox) test (E), *p <* *0.05, **p <* *0.01, ****p <* *0.0001.

Taken together, our results suggest that the IL-12 *T*. *gondii* strain that constitutively expresses GFP-dependent TMCs is necessary for host immunity against *T*. *gondii*. Thus, we hypothesized that conditionally deleting T-bet expression from TMCs would result in uncontrolled parasite replication and rapid host mortality. To test our hypothesis, we infected mice with a CD11c- and lysM-restricted deficiency of T-bet by using the CD11c- and lysM-Cre system (CD11c-*Tbx21*^−/−^; M-*Tbx21*^−/−^). Using infected M-*Tbx21*^−/−^ mice, we observed that conditional deletion of T-bet from monocytes, macrophages, and neutrophils did not result in reduced IL-12, increased parasite burden, or quicker host mortality during infection (Fig. 4C and data not shown). Additionally, infected CD11c-*Tbx21*^−/−^ mice demonstrated a significant reduction of T-bet expression by TMCs, yet there was no significant defect in IL-12 production or the recruitment of TMCs to the site of infection at day 5 PI (Fig. 4A–C). Strikingly, conditionally deleting T-bet from CD11c^+^ cells resulted in a significant increase of parasite burden compared with T-bet sufficient mice at the site of infection (Fig. 4D). Lastly, our data show that intrinsic T-bet expression by TMCs plays a role in host survival during *T*. *gondii* infection (Fig. 4E). Our results establish that an IL-12–mediated subpopulation of T-bet–dependent TMCs is linked to parasite clearance and host survival during acute *T*. *gondii* infection.

## Discussion

To our knowledge, this is the first study to demonstrate a role for T-bet in myeloid cell–mediated host defense during intracellular pathogen infection. Our findings demonstrate that T-bet is highly expressed in TMCs during infection, TMCs are associated with parasite clearance, and TMCs play a role in host survival during *T*. *gondii* infection. Taken together, these data indicate that TMCs are a novel subpopulation of T-bet–expressing myeloid cells that appear to play a role in host defense against intracellular pathogens.

Previous reports have shown that DCs express T-bet in response to IFN-γ (28). T-bet expression in DCs has been shown to be important in regulating cytokine and chemokine production and for their ability to prime naive CD4^+^ T cells (24–26). Hence, we anticipated that T-bet^+^ DCs would be critical for host resistance against *T*. *gondii*. Surprisingly, we observe limited T-bet expression in DCs, monocytes, macrophages, and neutrophils on days 0, 3, 5, and 8 PI. Collectively, these data illustrate that T-bet plays a very limited role in DC-, macrophage-, monocyte-, and neutrophil-mediated host defense against *T*. *gondii*.

Monocytes have been established to be critical for both acute and long-term host defense against *T*. *gondii* (29, 30). Additionally, numerous studies have shown that Ly6C^+^ monocytes have the capacity to differentiate into CD11c^+^ DCs during inflammatory conditions (30–34). Moreover, it has been shown during both *Leishmania major* and *T*. *gondii* infection that Ly6C^hi^ monocytes downregulate Ly6C and upregulate CD11c (30, 34). Biswas et al. (30) were able to further differentiate Ly6C^int-neg^ myeloid cells as Ly6C^int^CD11c^+^MHCI^+^MHCII^+^ and Ly6C^−^CD11c^+^F4/80^+^TREM2^+^, describing them to take on more DC-like characteristics and have elevated phagocytic capacity, respectively. Hence, it is possible that the Ly6C^lo^CD11c^+^MHCII^−^ TMCs we describe in this study could be differentiated from Ly6C^hi^ monocytes that have downregulated Ly6C and upregulated CD11c; however, based on our gating strategy that excludes F4/80^+^ and MHCII^+^ cells, it is unlikely that TMCs are one of the previously described populations observed during *T*. *gondii* or *L*. *major* infections. At this point, we do not have evidence indicating that TMCs are not monocyte derived, nor do we have contradictory data. Additional studies are underway to identify the precursor cells of TMCs, which we believe will significantly add to our knowledge of the role of T-bet in myeloid cell–mediated immunity.

Myeloid cell–mediated host defense is indispensable for *T*. *gondii* clearance (1). Innate recognition of *T*. *gondii* results in rapid IL-12 production, which largely comes from cDC1s (22, 23, 35). IL-12 is critical for the early activation and IFN-γ production of ILCs during intracellular microbial infection (36). Surprisingly, our results show contradictory findings, indicating that treating *Tbx21*^−/−^ mice with IL-12 was sufficient to reduce parasite burden on day 5 of infection, but insufficient to prevent rapid host mortality. Previous reports by our laboratory and others have shown that *T*. *gondii*–infected *Tbx21*^−/−^ mice retain the capacity to generate NK- and T cell–derived IFN-γ, and thus it is unlikely that T-bet–deficient animals with or without IL-12 treatment succumbed to infection due to the absence of IFN-γ (7, 16, 17). A potential explanation for these results stems from Harms Pritchard et al. (16), who showed that the absence of T-bet from T cells results in reduced expression of CD11a, Ly6C, and CXCR3, leading to fewer effector T cells present at secondary sites of infection, suggesting that loss of intrinsic T-bet expression by T cells is necessary for acute host resistance. An alternative explanation could be that although we show that IL-12 is necessary for the induction of CD11c^+^MHCII^−^ myeloid cells, the lack of intrinsic T-bet within these cells could result in a defect in their associated protective role for host defense against *T*. *gondii*. Most likely, an interplay of these two potential mechanisms may be influencing the acute host response against intracellular pathogens.

Along with myeloid cells being a critical source of IL-12 during infection, Sturge et al. (37, 38) demonstrated that *T*. *gondii* infection of *Rag2*^−/−^*γc*^−/−^ mice resulted in the appearance of IFN-γ^+^ cells and the induction of IFN-γ at significantly higher levels than WT mice, suggesting that myeloid cell–derived IFN-γ is sufficient for acute resistance against intracellular pathogen infection. IFN-γ recognition by myeloid cells is necessary for the initiation of IFN-γ–inducible genes, which mediate parasite elimination by tryptophan degradation, inhibition of metabolic enzymes, depletion of arginine, and disruption of the parasitophorous vacuole (11, 12, 39–46). Our results have added to the known role of myeloid cell–mediated host defense with the identification of TMCs, a T-bet–dependent myeloid cell population, which is critical for the clearance of the ubiquitous intracellular protozoan parasite, *T*. *gondii*. Furthermore, we have identified an additional IL-12 pathway that is essential for mediating myeloid cell–dependent host immunity.

## Supplementary Material

Supplemental Figures 1 (PDF)
