# Supplementary material for: IL-12 Mediates T-bet–Expressing Myeloid Cell–Dependent Host Resistance against Toxoplasma gondii
Source: Immunohorizons. 2024 Apr 30;8(4):355–62. doi: 10.4049/immunohorizons.2400029 (PMC11066714; doi:10.4049/immunohorizons.2400029)
Supplement: Supplemental Figures 1 (PDF) [file IH_2400029_Supplemental_1.pdf]

Supplemental Figure 1

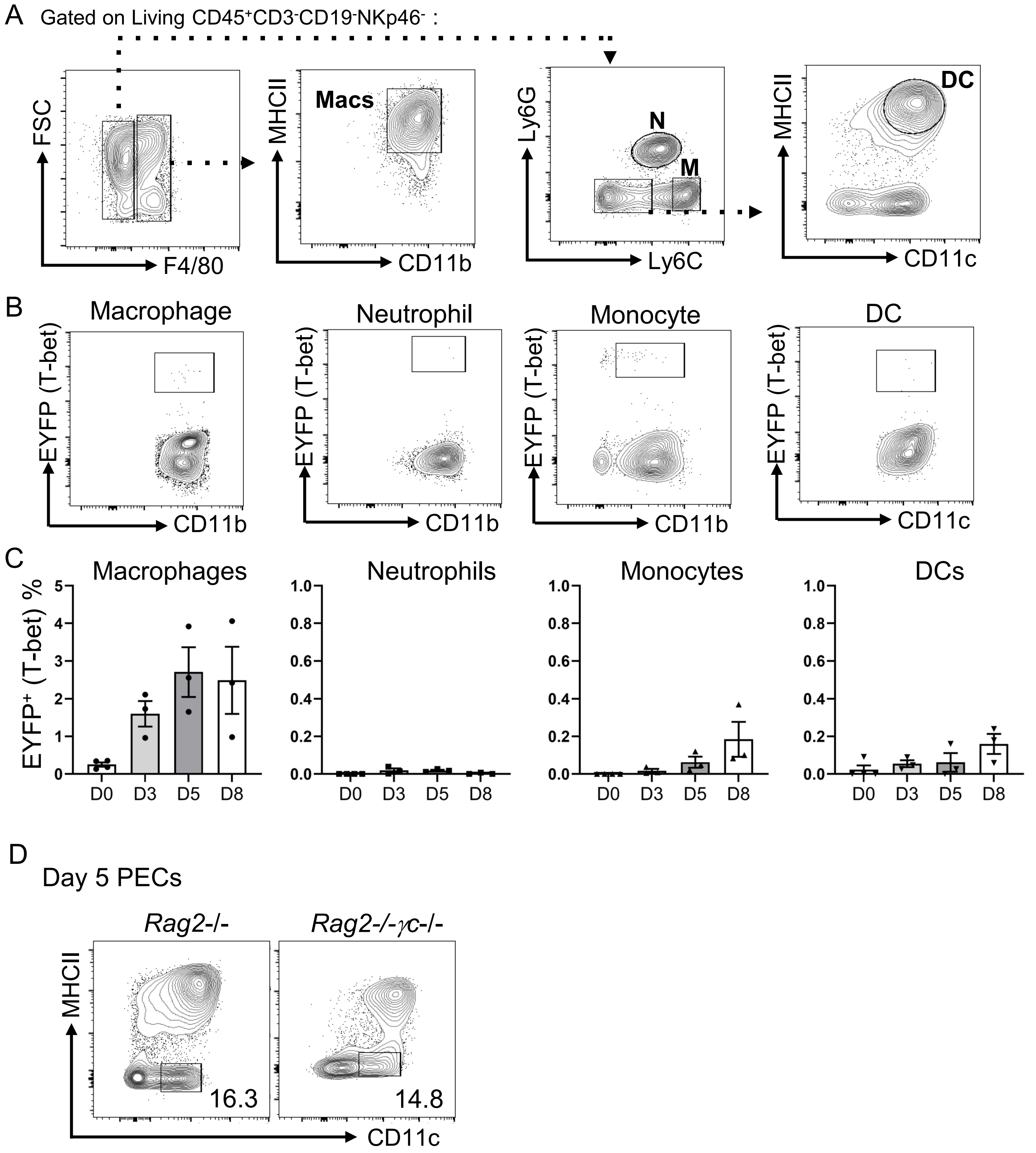

Supplemental Figure 1. T-bet expression in myeloid cells during *T. gondii* infection (**A-C**) Tbx21-EYFP mice were infected i.p. with 20 cysts and peritoneal myeloid cells were evaluated for EYFP expression. (A) Representative contour plots of $CD3^-CD19^-NKp46^-$ (lineage; Lin) macrophages (Macs), neutrophils (N), monocytes (M), DCs on day 5 p.i. (B) Representative contour plots of EYFP (T-bet) expression from (A), and (C) average frequencies of Lin^-EYFP^+ myeloid cells were analyzed on days 0, 3, 5, and 8 p.i. (**D**) *Rag2*$^{-/-}$ and *Rag2*$^{-/-}$*γc*$^{-/-}$ mice were infected i.p. with 20 cysts of *T. gondii*. Representative contour plots of $CD45^+F4/80^-$ $CD3^-CD19^-Ly6G^-NKp46^-Ly6C^{Lo}$ TMCs from *Rag2*$^{-/-}$ and *Rag2*$^{-/-}$*γc*$^{-/-}$ mice PECs that were harvested on day 5 post-infection. Error bars, SEM.

Supplemental Figure 2

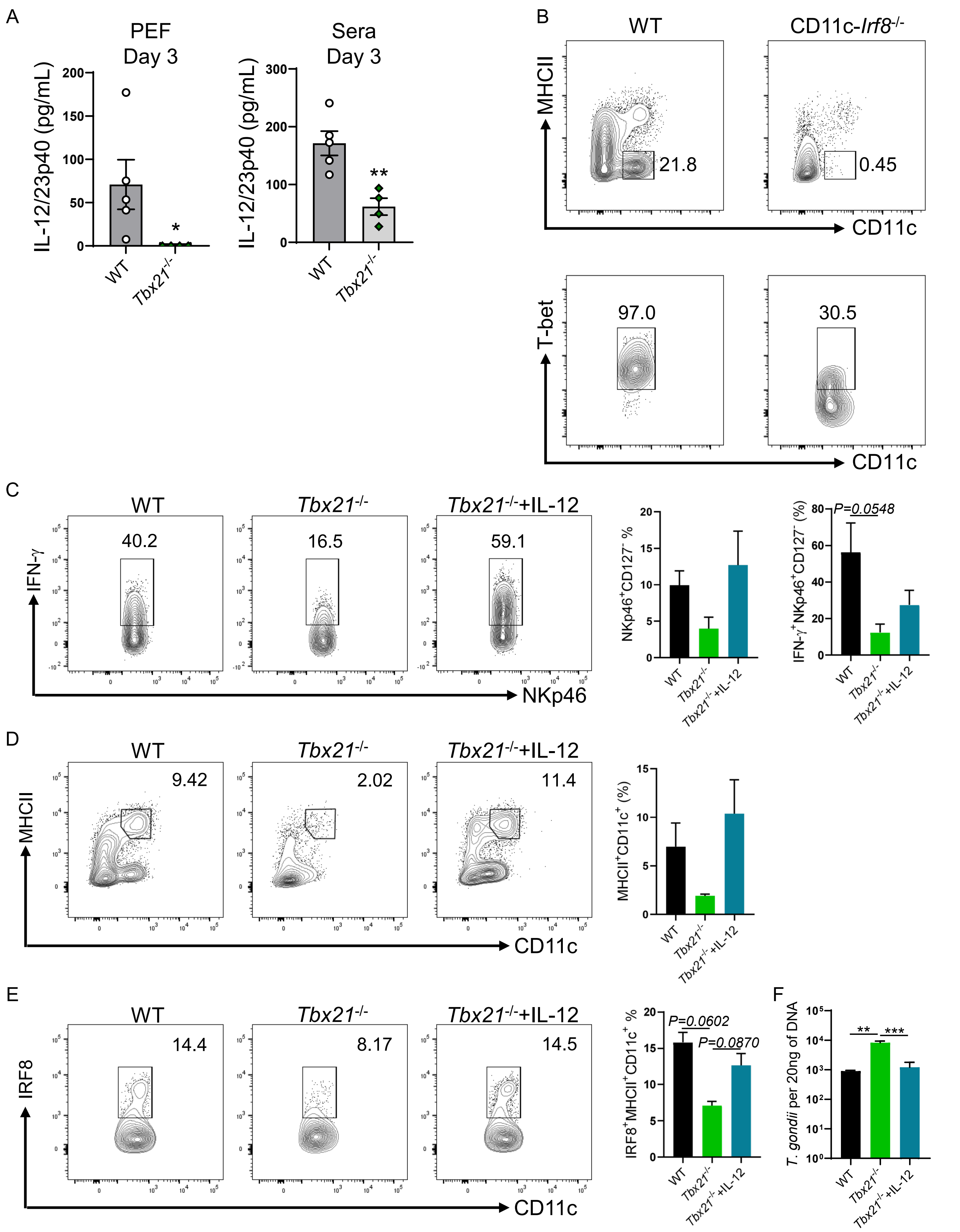

Supplemental Figure 2. TMCs are regulated by cDC1s (**A**) IL-12/23p40 analysis by ELISA of serum and PEF in mice following *T. gondii* infection on day 3 p.i. (**B**) WT and CD11c-*Irf8*$^{-/-}$ mice were infected i.p. with 20 cysts of *T. gondii*. Representative contour plots of CD45^{+}F4/80^{-}Lin (CD3^{-}CD19^{-}Ly6G^{-})$^{-}$ NKp46^{-}Ly6C^{Lo} TMCs and their T-bet expression from WT and CD11c-*Irf8*$^{-/-}$ mice PECs that were harvested on day 5 p.i. (**C-E**) *Tbx21*$^{-/-}$ mice were i.p. infected with *T. gondii* and treated with or without IL-12. (C) Representative contour plots and the average frequencies of CD127^{-}NKp46^{+} and CD127-NKp46^{+}IFN-γ^{+} NK cells in the PECs were analyzed on day 2 following infection and cytokine treatment. (D) Representative contour plots and average frequencies of Lin^{-}CD11c^{+}MHCII^{+} DCs in the PECs were analyzed on day 5 following infection and cytokine treatment. (E) Representative contour plots and average frequency of Lin^{-}CD11c^{+}MHCII^{+}IRF8^{+} DCs in the PECs were analyzed on day 5 following infection. (**F**) Parasite burden was assessed from the PECs in *T. gondii*-infected WT, and *Tbx21*$^{-/-}$ with or without IL-12 treatment on day 5 p.i. by qPCR. Statistical analyses were done using unpaired *t*-test analysis of individual groups or one-way ANOVA with a post Tukey's multiple comparison test, $*P < 0.05$, $**P < 0.01$, $***P < 0.001$. Error bars, SEM.

Supplemental Figure 3

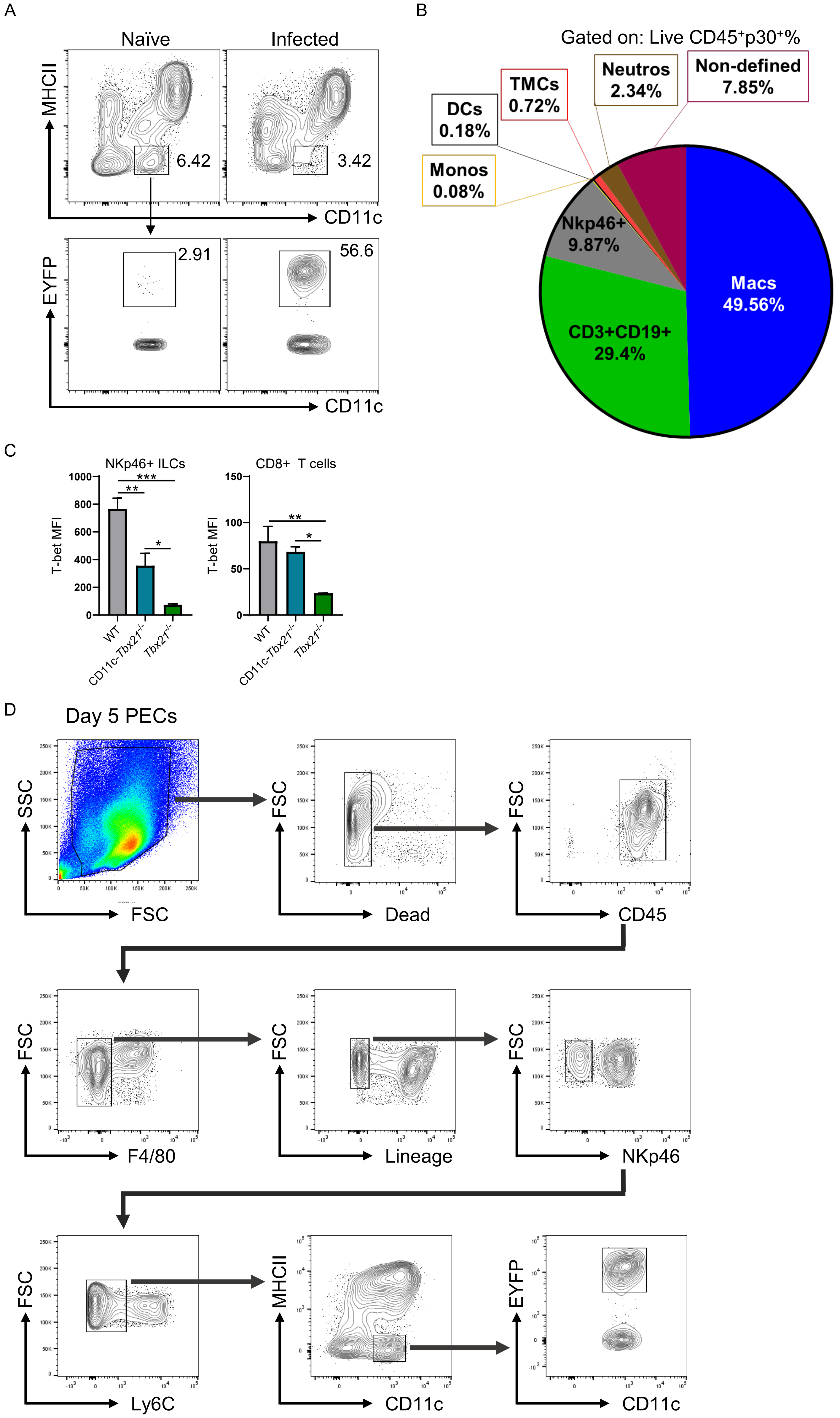

Supplemental Figure 3. Representative TMC gating strategy (**A**) Tbx21-EYFP mice were infected i.p. with 20 cysts of *T. gondii*. Representative contour plots of CD45$^+$F4/80$^-$Lin (CD3$^-$CD19$^-$Ly6G$^-$)$^-$ NKp46$^-$Ly6C^{Lo} TMCs and their EYFP (T-bet) expression from naïve and infected Tbx21-EYFP mouse spleens that were harvested on day 5 p.i. (**B**) WT mice were i.p. infected with 20 cysts. Representative pie chart with the frequencies of Live CD45$^+$p30$^+$ Nkp46$^+$ ILCs, T cells, B cells, monocytes, macrophages, DCs, neutrophils, and TMCs from the peritoneum on day 5 p.i. (**C**) WT, CD11c-*Tbx21*$^{-/-}$, and *Tbx21*$^{-/-}$ mice were i.p. infected with 20 cysts. (C) The MFI of Nkp46$^+$ and CD8$^+$ subsets T-bet expression in the PECs was analyzed on day 5 p.i. (**D**) TMCs in the peritoneal cavity were defined as CD45$^+$F4/80$^-$Lin (CD3$^-$CD19$^-$Ly6G$^-$)$^-$ NKp46$^-$Ly6C^{Lo}CD11c$^+$MHCII$^-$ cells. Statistical analyses were done using a one-way ANOVA with a post Tukey's multiple comparison test, *$P < 0.05$, **$P < 0.01$, ***$P < 0.001$. Error bars, SEM.

Supplemental Table I

Model: / Cell type	B6	$Rag2^{-/-}\gamma c^{-/-}$	$Tbx21^{-/-}$	T-bet expression seen in Tbx21-EYFP mice
Neutrophils	Yes (1, 2)	Yes	Yes [unpublished observation]	Limited
Monocytes	Yes (3)	Yes	Yes-Requires ILC-derived IFN-γ to be sustained (4)	Limited
DCs	Yes (5, 6)	Yes	Yes-Requires ILC-derived IFN-γ to be sustained (6)	Limited
Macrophages	Yes (7)	Yes	Yes [unpublished observation]	Low
ILC1s	Yes (4)	No	No (4, 6)	Not tested
NKs	Yes (8)	No	Yes (6)	High [unpublished observation]
T cells	Yes (9)	No	Yes (10, 11)	High [unpublished observation]
B cells	Yes (12)	No	Yes [unpublished observation]	Not tested

Supplemental Table I Summary of critical immune cells during acute *T. gondii* infection The table summarizes the known myeloid cells and lymphocytes that have been established to play a critical role in host defense during acute toxoplasmosis in B6, $Rag2^{-/-}\gamma c^{-/-}$, and $Tbx21^{-/-}$ mouse models. Additionally, we summarize our findings of T-bet expression in neutrophils, macrophages, monocytes, DCs, NKs, T cells, and B cells from *T. gondii* infected Tbx21-EYFP mice.

References:

1. Dunay, I. R., A. Fuchs, and L. D. Sibley. 2010. Inflammatory monocytes but not neutrophils are necessary to control infection with Toxoplasma gondii in mice. Infect Immun 78: 1564-1570.
2. Sturge, C. R., A. Benson, M. Raetz, C. L. Wilhelm, J. Mirpuri, E. S. Vitetta, and F. Yarovinsky. 2013. TLR-independent neutrophil-derived IFN-gamma is important for host resistance to intracellular pathogens. Proc Natl Acad Sci U S A 110: 10711-10716.
3. Robben, P. M., M. LaRegina, W. A. Kuziel, and L. D. Sibley. 2005. Recruitment of Gr-1+ monocytes is essential for control of acute toxoplasmosis. J Exp Med 201: 1761-1769.
4. Klose, C. S., M. Flach, L. Mohle, L. Rogell, T. Hoyler, K. Ebert, C. Fabiunke, D. Pfeifer, V. Sexl, D. Fonseca-Pereira, R. G. Domingues, H. Veiga-Fernandes, S. J. Arnold, M. Busslinger, I. R. Dunay, Y. Tanriver, and A. Diefenbach. 2014. Differentiation of type 1 ILCs from a common progenitor to all helper-like innate lymphoid cell lineages. Cell 157: 340-356.
5. Reis e Sousa, C., S. Hieny, T. Scharton-Kersten, D. Jankovic, H. Charest, R. N. Germain, and A. Sher. 1997. In vivo microbial stimulation induces rapid CD40 ligand-independent production of interleukin 12 by dendritic cells and their redistribution to T cell areas. J Exp Med 186: 1819-1829.
6. López-Yglesias, A. H., E. Burger, E. Camanzo, A. T. Martin, A. M. Araujo, S. F. Kwok, and F. Yarovinsky. 2021. T-bet-dependent ILC1- and NK cell-derived IFN-γ mediates cDC1-dependent host resistance against Toxoplasma gondii. PLoS Pathog 17: e1008299.
7. Park, J., and C. A. Hunter. 2020. The role of macrophages in protective and pathological responses to Toxoplasma gondii. Parasite Immunol 42: e12712.
8. Denkers, E. Y., R. T. Gazzinelli, D. Martin, and A. Sher. 1993. Emergence of NK1.1+ cells as effectors of IFN-gamma dependent immunity to Toxoplasma gondii in MHC class I-deficient mice. J Exp Med 178: 1465-1472.
9. Hunter, C. A., C. S. Subauste, V. H. Van Cleave, and J. S. Remington. 1994. Production of gamma interferon by natural killer cells from Toxoplasma gondii-infected SCID mice: regulation by interleukin-10, interleukin-12, and tumor necrosis factor alpha. Infect Immun 62: 2818-2824.
10. López-Yglesias, A. H., E. Burger, A. Araujo, A. T. Martin, and F. Yarovinsky. 2018. T-bet-independent Th1 response induces intestinal immunopathology during Toxoplasma gondii infection. Mucosal Immunology.
11. Harms Pritchard, G., A. O. Hall, D. A. Christian, S. Wagage, Q. Fang, G. Muallem, B. John, A. Glatman Zaretsky, W. G. Dunn, J. Perrigoue, S. L. Reiner, and C. A. Hunter. 2015. Diverse roles for T-bet in the effector responses required for resistance to infection. J Immunol 194: 1131-1140.
12. Frenkel, J. K., and D. W. Taylor. 1982. Toxoplasmosis in immunoglobulin M-suppressed mice. Infect Immun 38: 360-367.
